# A qualitative investigation of tobacco use and cessation experiences among people living with HIV in Botswana

**DOI:** 10.1186/s12982-026-02813-1

**Published:** 2026-09-18

**Authors:** Megan E. Mansfield, Florence Bada, Selebaleng V. Mokonopi, Natalia Blanco, Kaizer Ikgopoleng, Lillian Okui, Carlo DiClemente, Roy Tapera, Bontle Mbongwe, Jessica Magidson, Seth Himelhoch, Manhattan Charurat

**Affiliations:** 1 Division of Epidemiology and Prevention, Institute of Human Virology, University of Maryland School of Medicine, 725 W. Lombard Street, Baltimore, MD 21201 USA; 2 Botswana University of Maryland Medicine Health Initiative, Gaborone, Botswana; 3 Department of Psychology, University of Maryland Baltimore County, Catonsville, MD USA; 4 School of Public Health, University of Botswana, Gaborone, Botswana; 5 Department of Psychology and the Center for Substance Use, Addiction & Health Research (CESAR), University of Maryland, College Park, MD USA; 6 Department of Psychiatry and Behavioral Neuroscience, The University of Chicago, Chicago, IL USA

**Keywords:** Tobacco use, Smoking cessation, Botswana, PLWH, Qualitative research, Thematic analysis

## Abstract

**Background:**

Tobacco use is a leading cause of premature mortality among people living with HIV (PLWH), especially in Botswana, where high smoking rates coincide with successful HIV control. There is a need for evidence-based interventions to support smoking cessation among PLWH, but research on smoking cessation in Botswana is limited. Therefore, to address this gap, a qualitative study was conducted in Botswana to understand the experiences of PLWH who smoke and identify barriers and facilitators to quitting in preparation for a larger implementation trial.

**Methods:**

In 2024, 36 interviews were conducted with adult patients who smoke from five HIV care sites in Botswana. Participants were purposively sampled by cessation outcome: successfully quit (*n* = 13), returned to use (*n* = 8), and did not quit (*n* = 15). The interviews aimed to gather insights into smoking cessation barriers and facilitators, including behavioral and motivational factors. The interviews were recorded, transcribed, and translated from Setswana to English, then were analyzed using a hybrid deductive-inductive thematic analysis approach.

**Findings:**

Results showed notable differences in the reasons for starting and continuing to smoke. Despite strong motivations to quit, such as reducing financial, health, and social burdens, participants encountered obstacles such as withdrawal symptoms and life stressors. Participants also identified various strategies to aid quitting like eating candies and attending church, and expressed readiness to use medical services such as cessation medications.

**Conclusion:**

Overall, participants expressed a strong desire for interventions to help them quit smoking. Effective interventions could focus on managing withdrawal symptoms and utilizing existing patient motivations and social supports.

*Clinical Trial Registration* NCT05694637 Registered on 7 December 2022 on clinicaltrials.gov, https://clinicaltrials.gov/search? locStr=Botswana&country=Botswana&cond=Smoking%20Cessation&intr=SBIRT.

**Supplementary Information:**

The online version contains supplementary material available at https://doi.org/10.1186/s12982-026-02813-1.

**Implications** Tobacco smoking is a major health risk in Botswana, and more so among people living with HIV. In order to identify contextuallyappropriate evidence-based interventions and relevant adaptations for promoting smoking cessation, a formative understanding of thispopulation’s experiences with smoking cessation is needed. Relevant approaches and strategies to support smoking cessation among peopleliving with HIV in Botswana include pharmacological and motivational communication strategies.

**Summary of research** The use of pharmacological and motivational communication strategies may be well suited for supporting smoking cessation among peopleliving with HIV in Botswana.

## Introduction

With the increased availability of human immunodeficiency virus (HIV) treatment, focus has shifted from concerns about the life expectancy of people living with HIV (PLWH) to their quality of life [1]. This shift includes health risks such as cardiovascular diseases, certain cancers, and lung diseases including bacterial pneumonia [2–4]. As compared to the general population, a higher proportion of PLWH smoke [5–11] and smoking tobacco is a leading cause of morbidity and premature mortality among PLWH [12–22]. Despite the desire of many PLWH to quit smoking, they require assistance to do so [9, 23–24]. This phenomenon is particularly salient in Botswana, an upper middle-income country that has achieved epidemic control of HIV but has high prevalence of cigarette smoking that is expected to increase [25–27]. For example, in 2017, only 7% of adults in Botswana who attempted to quit smoking were successful [27]. Numerous strategies supported by research have been identified to help support smoking cessation. These include various counseling approaches [28], tailored behavioral interventions [29–33], nicotine replacement therapy [34], and other pharmacotherapies [34–37]. However, it is essential that smoking cessation efforts are tailored to address the specific needs and circumstances of individual clients [33, 38].

In order to implement a tobacco cessation program for PLWH in Botswana that is both contextually appropriate and sustainable, it is crucial to identify the barriers and facilitators specific to this population. To address this gap, we aimed to collect formative information on the barriers and facilitators to smoking cessation through an in-depth exploration of the experiences of PLWH in Botswana who were interested in quitting smoking. This formative research is necessary for designing and studying the effectiveness of context appropriate procedures for counseling PLWH in Botswana on smoking cessation. The findings of this qualitative study may therefore inform implementation and adaptation of smoking cessation interventions to fit the cultural and practical context of Botswana.

## Methods

This study used a descriptive qualitative design to explore the experiences of PLWH in Botswana regarding tobacco cessation. We used a semantic constructionist approach to prioritize participants’ stated experiences with tobacco use and cessation, as well as broader healthcare engagement. The data were collected as part of the control phase of the Botswana Smoking Abstinence Reinforcement Trial (BSMART) [38] and reporting of this study was guided by the Consolidated Criteria for Reporting Qualitative Research (see Supplementary Materials 1) [39].

### Data collection procedures

These data were collected by local research assistants with training in qualitative and quantitative data collection from five randomly selected HIV care facilities in Botswana out of the 15 participating in BSMART [38]. These facilities included one referral hospital (Nyangabgwe Referral Hospital), two primary hospitals (Sefhare and Tutume Primary Hospitals), and two clinics (Area W and Masego Clinics), representing a mix of urban and rural healthcare settings. These facilities offer HIV care and treatment services to a total of 13,451 PLWH enrolled in the HIV Clinics at the time of study enrollment.

BSMART participants were purposefully recruited by the study research assistants to participate in an interview during their Week 24 clinic visit. Inclusion criteria included living with HIV and engaged in HIV care, currently smoking, aged 18 years or older, and willing/able to provide informed consent in English or Setswana. Further, participants were purposefully sampled based on their smoking cessation behavior stratified within their health facility (see Table 1).

We attempted to conduct interviews with an equal number of participants who successfully quit smoking, did not quit, and returned to use (i.e., stopped smoking but resumed again) across all five sites. Additionally, due to the small number of women enrolled in the BSMART study, all women-identified participants were given the opportunity to participate regardless of the state of sampling.

Participants completed the interview in a private space. Interviews were audio recorded and conducted by study research assistants who had training in qualitative interviewing and were fluent in the local languages. Audio recordings were simultaneously transcribed verbatim and translated into English for analysis by the research assistants. The sample size was sufficient as saturation was achieved.

### Data analysis

Data analysis followed a hybrid deductive-inductive thematic analysis [40].Thematic analysis is a flexible analytic procedure that focuses on the experiences of participants in their own words [40–41]. Deductive approaches build from existing theory or frameworks [42] whereas inductive approaches allow for themes or patterns to emerge organically from the data [43]. A hybrid approaches uses components of both and allowed us to identify key themes related to both motivations for tobacco use and facilitators and barriers to smoking cessation among PLWH who smoke in Botswana. Specifically, the analytic team began with a more deductive approach. After reading the transcript to familiarize themselves with the data, the team developed an initial codebook with codes primarily identified based on the interview guides (see Supplementary Materials 2) and aims identified by the parent study [38]. However, to fully capture the experiences discussed by participants, the analytic team identified additional inductive codes through iterative refinement of the codebook. These inductive topics include initial experiences with tobacco use and impact of their HIV diagnosis. The refined codebook was pilot tested on a subset of transcripts (k = 6) using ATLAS.ti [44] and adjusted further to improve interrater reliability. Each transcript (*n* = 36) was coded by two coders using the finalized codebook (see Supplementary Materials 3). Interrater reliability was assessed qualitatively using ATLAS.ti, and disagreements were discussed until consensus was reached. After coding was completed, themes were organized using axial coding, and key themes are presented with support from participant quotations. Throughout the coding and thematic analysis process, the coders engaged with local members of the research team to promote culturally sensitive interpretations.

## Findings

We conducted 36 semi-structured interviews from January – March 2024, during which participants were asked about their experiences with tobacco use and smoking cessation (e.g., what prompted you to quit smoking in the past?). Most participants identified as men (*n* = 27; 75%) and there was approximately equal representation of participants by smoking cessation outcome and facility (see Table 1), though the returned to use group was the most challenging to recruit given few participants met the criteria (*n* = 8; 22%).

**Table 1:** Participant sampling parameters and final sample by facility and smoking cessation behavior

	Facility 1	Facility 2	Facility 3	Facility 4	Facility 5	Total
**Stratified Sampling Parameters (** ***n* = 30)**						
Quit	2	2	2	2	2	10
Returned to use	2	2	2	2	2	10
Did not quit	2	2	2	2	2	10
Total	6	6	6	6	6	30
**Final Sample (*n*** **= 36)**						
Quit	3	2	3	3	2	13
Returned to use	2	1	2	1	2	8
Did not quit	2	3	5	2	3	15
Total	7	6	10	6	7	36

Participants were mostly single and never married (75%) and their age ranged from 23 to 73 (Median = 47). Most participants reported completing junior secondary-level schooling (47%), followed by primary school (25%) with some reporting senior secondary (8%), tertiary (8%), or none (11%). 42% of participants were employed and 36% were unemployed.

Four primary themes emerged across three topic areas that aligned with our primary aim of gathering in-depth information to inform the adaptation and implementation of evidence-based interventions to support smoking cessation among PLWH in Botswana (Table 2). This included the identification of barriers, facilitators, and identified strategies for smoking cessation.

**Table 2:** Identified topic areas, primary themes, and sub-themes

Primary theme	Sub-theme
Smoking motivations	Initial curiosity
	Current stress and dependency
Barriers to smoking cessation	Biophysiological barriers
	Environmental stressors
	Impact of HIV status inconclusive
Reasons to quit	External pressures
	Internal motivations
Quitting strategies	Introduce replacements for cigarettes
	Make changes to environment
	Engage social support
	Seek additional treatments

### Smoking motivations

#### Initial curiosity

Most participants described being younger when they started smoking and feeling curious about smoking. For example, one participant who was able to quit but resumed smoking stated,

“My smoking started was due to me hanging out with my friends who smoked and being curious and interested in smoking until the habit stuck” (49 year old man from Site 3).

Another participant described how when they were younger cigarettes seemed like fun,

“Initially it was just playing and fun, we thought cigarette was fashionable, and thought when a person was holding a cigarette, they felt big and nice” (49 year old man from Site 5).

Often this curiosity about smoking was informed by observing other people in their lives’ smoking – including friends and family. One participant who did not quit described,

“It was just an issue of trying to look cool, as sometimes as a man you should be seen with a pack of cigarettes when out at night. I wasn’t necessarily forced to. It was just me trying to fit in society and be accepted by my friends” (47 year old man from Site 1).

Sometimes participants experienced social pressures to try smoking, but few explicitly stated they were pressured to smoke. For example, one participant described,

“It was peer pressure. I was just impressed that others are smoking” (46 year old man from Site 1).

Another participant stated,

“It was just fun when see my peers smoking and thought it was something beneficial” (70 year old man from Site 1).

#### Current stress and dependency

Current or recent smoking motivations were related to various environmental stressors and physical dependency. Participants described feelings of anxiety, depression, and boredom motivating their smoking. For example, one participant who did not quit described,

“When I smoke, it’s because I believe it’s a form of self-medication. At first it was all about peer pressure since the people I hung out with also smoked, I decided to join them so that we match and become one, but now it’s no longer about that. I use it to manage my anger and depression” (47 year old man from Site 1).

Another participant similarly stated that they had

“hoped smoking would help me manage my life stresses” (36 year old woman from Site 5).

Additionally, many participants noted that they tended to smoke when they were also drinking alcohol:

“After I started drinking alcohol, I then started smoking” (40 year old man from Site 3).

Participants also spoke specifically about festive seasons being a difficult time to avoid smoking as they are often hanging out with friends and going to bars:

“Whenever I am with my peers and they are drinking alcohol, I start smoking” (59 year old woman from Site 3).

Participants also described various physiological reasons for smoking tobacco. Some described increased energy or clarity of mind after smoking. One participant noted that smoking is

“now all about uplifting my spirits whenever I am feeling low” (43 year old man from Site 5).

Participants also described continuing to smoke to reduce discomforts like pain, headaches, nose bleeds, and cravings. One participant described,

“Initially I smoked for nose-bleeding and later when it is already in your body, you can’t leave easily, sometimes when you wake up in the morning you feel your head is just heavy and not functioning properly and you are supposed to take a puff so that you can think properly” (48 year old man from Site 5).

### Barriers to smoking cessation

#### Biophysiological barriers

The physiological side effects of dependency both facilitated continued smoking and impeded quit attempts. For example, one participant described

“getting dizzy and got headaches as well. I couldn’t concentrate at work when I didn’t smoke” (23 year old man from Site 5).

Another participant described stresses associated with holidays making it difficult for them to quit,

“this Christmas holiday is the one that killed me as I had a lot of stress…and was smoking to relieve stress” (48 year old man from Site 5).

#### Environmental stressors

Participants described specific places or situations where they tended to smoke more, such as at work, drinking alcohol, or socializing with friends. Being in these spaces or situations made it difficult to resist smoking. For example, one participant stated,

“staying with someone who smokes because it makes it difficult for me to stop as we spend most of the time together and they smoke most of the time. That is the only challenge” (29 year old woman from Site 4).

#### Impact of HIV status inconclusive

Notably, there was no consensus among the participants regarding the impact of their HIV status on their smoking behaviors. Some participants described increases in smoking following their HIV diagnosis due to the stress and anxiety the diagnosis initially caused them. One participant described that their HIV diagnosis

“affected me a little bit. Then I smoked more, but I ended up accepting the situation” (30 year old man from Site 3).

Another participant noted

“Since I have known about my HIV status, sometimes you feel down and low and then try to cheer yourself up by smoking. So since I’ve known that I am HIV positive, I smoke more than before” (43 year old man from Site 5).

However, other participants described that their HIV diagnosis resulted in a reduction of tobacco use due to an increased concern for their overall health. For example, one participant stated that

“After I was diagnosed with this disease [HIV], there was a time it really debilitated me, and I did not smoke” (70 year old man from Site 1).

But many participants described their HIV diagnosis as having no impact on their smoking behaviors – they smoked the same amount before and after the diagnosis:

“No, I think it [smoking] has nothing to do with HIV. I have accepted my situation a long time back, so it has nothing to do with it [smoking]” (42 year old man from Site 4).

### Reasons to quit

#### External pressures

Participants described peer pressure from family and friends. One participant described how they believe living with their family would have created an environment not conducive to smoking:

“If I had stayed with my children and their mother in one place, I wouldn’t be smoking tobacco, even when I used to stay with them, I would not smoke at home as I didn’t want them to see me smoking, I would never come home with cigarette[s] because I knew I didn’t want the children to see what I am doing” (49 year old man from Site 5).

Ultimately, participants felt uncomfortable or embarrassed about their tobacco dependency. One participant described,

“It [smoking] is also very embarrassing especially as a woman… Smoking is very dangerous and it was also embarrassing for me. Imagine during weddings and parties when I wanted to smoke, I had to go hide. When I was done smoking, I reeked of cigarettes… Even church right now I attend church. I previously wouldn’t go to church because I was afraid they would say I reek of cigarette smoke. I was just from church yesterday” (54 year old woman from Site 1).

Participants also discussed the financial cost of smoking. One participant described realizing that “smoking used a lot of my money” (41 year old man from Site 2) Similarly, another participant noted,

“My smoking habit kept me broke all the time. I was never able to have any pocket money” (31 year old man from Site 3).

#### Internal motivations

Personal commitments to quitting smoking were emphasized as incredibly important among many participants. They were quite confident that a strong personal conviction was the most important factor in successfully quitting smoking. One participant who reported successfully quitting during the BSMART study described that

“There are no services or resources that I can say helped me. The only thing that helped me was, I had already told myself, personally, that I want to stop” (41 year old man from Site 2).

Another participant who successfully quit noted,

“I managed because I was determined to stop smoking and I told myself, despite that challenge, I will have to stop and will not be tempted to smoke” (29 year old woman from Site 4).

Overall, many participants believed that the appropriate discipline or mind set was all they required to quit successfully:

“It is because of my commitment that I am going to stop” (46 year old man from Site 1).

Another participant who quit but ultimately resumed smoking stated,

“I trust myself a lot. I trust myself that I will manage to quit. I haven’t and will never give up” (55 year old man from Site 1).

Participants also discussed the importance of health education and learning about the risks smoking poses to their health, especially considering their HIV status. For example, one participant who was able to quit but later resumed smoking noted that

“some of the reasons that make me want to quit is because of the information you have shared with me. As you can tell me on how when I have certain ailments, I shouldn’t smoke cigarettes and that I should either reduce or quit smoking. I totally agree with that” (55 year old man from Site 1).

Another participant described,

“There are a lot of smoking related diseases, and when you look at them, their outcomes are not good at all, like cancer, eish, when you think about it, you find that it is you who caused it by tobacco, it becomes painful” (46 year old man from Site 1).

Participants also brought up realizing that smoking was aggravating existing ailments and created additional concerns for other treatments they were receiving (e.g., TB treatment). For example, one participant noted

“I stopped because I was taking TB treatment” (70 year old man from Site 1) and another stated “I could see I will end up having TB because of tobacco” (55 year old woman from Site 4).

Further, some participants were concerned about the impact their smoking was having on other people in their lives (e.g., children).

### Quitting strategies

#### Introduce replacements for cigarettes

Many participants described replacing smoking with something else – like mint or candies:

“The chewing gums that are used to help stop smoking. I use those a lot” (29 year old woman from Site 4).

Another participant who successfully quit noted,

“When I craved [cigarettes], I ate some sweets to help with the craving” (63 year old woman from Site 3).

Additionally, participants described engaging in different activities (e.g., physical exercise) to keep them occupied. A few participants had used this strategy and found it beneficial, stating,

“If I could manage to maybe find somewhere to gym and also encourage others to gym so that we can try break the nicotine we have accumulated in our bodies” (47 year old man from Site 1).

#### Make changes to environment

Participants also discussed being conscientious of their environment and trying to frequent smoke-free environments (e.g., church) more often to avoid the temptation. Although they experienced a relapse in smoking, one participant described,

“I visited the church a lot. When I went there with the people that I was with, none of them smoked” (47 year old man from Site 1).

This strategy also included avoiding, when possible, other people who smoked:

“avoiding staying for long in places where people smoke, staying away from people who smoke” (29 year old woman from Site 4).

But this was a challenge for participants who lived with another person who smoked tobacco.

#### Engage social support

Family, friends, and co-workers were sources of support the participants discussed using to help their quitting efforts. One participant who successfully quit described,

“I have a friend who I am accountable to who I told when quitting smoking. Even up to now he makes follow ups and checks in to see if I haven’t gone back to smoking” (47 year old man from Site 5).

Another participant described how being around their family keeps them accountable to their quitting goal,

“I have a lot of support from my relatives. None of them really wants me to be smoking…I never smoke around them” (47 year old man from Site 1).

#### Seek additional treatments

Although participants discussed the potential utility of therapy or support groups, they were very interested in medications that could ease their symptoms. One participant noted,

“I wish there was something like a pill or injection prescribed to help me quit smoking” (23 year old man from Site 5) and another stated, “If there is a medication and is available may help” (23 year old man from Site 3).

As this is not a resource participants had previous experiences or opportunity, they were very interested:

“From the hospital I have heard there is a pill that can make me do better in quitting. My wish is, if it is available, I should be provided with it” (46 year old man from Site 3).

## Discussion

Botswana has achieved epidemic control of HIV, making it important to shift priorities from ensuring the survival of PLWH to promoting a high quality of life. Addressing health risk factors such as smoking, which is more common in PLWH than in the general population and a leading cause of morbidity and premature mortality among PLWH, is essential [25–27]. This is a public health issue that can be addressed using numerous research-supported strategies to improve smoking cessation [28–37]. Furthermore, implementation science research has demonstrated the importance of adapting and integrating contextually appropriate implementation strategies and adaptations for optimizing public health interventions, particularly in novel contexts [45].

To identify contextually relevant adaptations and implementation strategies for a smoking cessation intervention in Botswana, we conducted an exploratory qualitative research study. We interviewed 36 PLWH in Botswana to understand the barriers and facilitators to smoking cessation.

Results highlight that physical dependency on tobacco is a salient barrier to many PLWH who smoke in Botswana. Participants expressed concerns about fatigue, brain fog, headaches, and nose bleeds, which hindered their day-to-day functioning when attempting to quit smoking. Therefore, future efforts to support smoking cessation should explore mechanisms for addressing these concerns. One recommendation that received enthusiastic acceptance from participants was the idea of pharmacological intervention. There are various evidence-supported pharmacological interventions that support smoking cessation. For example, varenicline is considered the most effective pharmacological intervention for smoking cessation [35, 37] and various anti-depressants like bupropion have been shown to be more effective than placebos^34^. Nicotine replacement therapies in the form of nasal sprays, patches, gum, and inhalers have similarly successful effectiveness rates 2.1–3.1 times more effective than placebos [34]. The existing research supporting the effectiveness of these interventions and participants’ acceptability of them make pharmacological interventions a potentially contextually appropriate smoking cessation intervention for PLWH who smoke in Botswana.

Our analysis also revealed that participants are very motivated to quit for various reasons – including concerns about their health and social relationships. Therefore, motivational communication strategies may also be effective in this context. Existing research suggests that these approaches are effective for supporting smoking cessation [28]. For example, one study found higher rates of quitting among participants who received motivational interviewing interventions (16.9%) as compared to participants in the control group (14.2%) [46] and another study found modest effects of motivational interviewing on abstinence in a medical setting [47]. Although the effectiveness of these kinds of interventions varies based on the specific treatment and participants’ readiness to change, existing research suggests that these interventions are particularly effective for smoking and alcohol cessation [28].

Additionally, our analyses demonstrated that participants’ motivations for smoking likely change over time. Therefore, in implementing motivational communication strategies, it is critical that providers identify current motivations for quitting and continuing to smoke in order to counsel people effectively. It may be advisable to check in over time as these motivations may change. This may be even more important among PLWH or those vulnerable to HIV, as, based on our sample, HIV diagnosis has a variable impact on people and their smoking behaviors. Notably, after accepting the diagnosis, PLWH may not have the motivation to quit unless providers highlight potential health risks. Future research may benefit from the use of intensive longitudinal approaches, like experience sampling or daily diaries, to improve understanding of smoking cognitions and experiences as they – and co-occurring health conditions – shift over time. Overall, given some of the motivational states expressed by participants in this study, motivational communication interventions may be appropriate for use among PLWH who smoke in Botswana. However, future research is needed to examine the extent to which motivational communication interventions generalize to this specific context or how they may need to be further adapted.

### Limitations

Although informative, this study has some important limitations. The findings may not be generalizable to all people who smoke in Botswana. For example, although participants did not note a consistent effect of their HIV status on their smoking behaviors, this characteristic does distinguish them from people who smoke and are not living with HIV. Future research should investigate important differences among people who smoke in Botswana not living with HIV to ensure smoking cessation efforts are appropriate for them as well. Additionally, this study only represents the perspective of one group that was already enrolled in a research trial and willing to provide informed consent for an additional interview. Future research should include a broader range of stakeholders, such as community members, healthcare providers, clinic administrators, and representatives of the Ministry of Health. Finally, the exploratory nature of this research led to a breadth of topics being included as part of the interview data collection; however, these were all linked in this analysis to the specific aims of this study to inform intervention adaptation and implementation. Although the interviews were brief and this may have limited the depth of possible discussion, we still received rich information from participants. Future research may want to investigate more nuances to the smoking experiences of people in Botswana.

## Conclusion

Successful implementation of smoking cessation interventions for PLWH in Botswana requires consideration of both the existing research-supported interventions and the context and, most importantly, the fit between these two components. The findings from this study represent a critical step in identifying relevant approaches and strategies to support smoking cessation efforts among PLWH in Botswana. The insights gained from this study will inform the adaptation of smoking cessation interventions – including pharmacological and motivational communication strategies – to be implemented and evaluated in various HIV care and treatment facilities throughout Botswana as part of the BSMART study.

## Supplementary Information

Below is the link to the electronic supplementary material.

Supplementary Material 1.

Supplementary Material 2.

Supplementary Material 3.

## Data Availability

The data analyzed for the current study are not publicly available due to their sensitivity but are available from the corresponding author upon reasonable request. Data collection materials, including the interview guide, are included in the supplemental materials.
