# Supplementary material for: A qualitative investigation of tobacco use and cessation experiences among people living with HIV in Botswana
Source: Discov Public Health. 2026 Sep 18;23(1):1483. doi: 10.1186/s12982-026-02813-1 (PMC13588883; doi:10.1186/s12982-026-02813-1)
Supplement: Supplementary file 1 [file 12982_2026_2813_MOESM1_ESM.docx]

**Supplementary Materials 3: Final Codebook**

| **Color** | **Code** | **Definition** | **Notes** |
| --- | --- | --- | --- |
| Green | Facilitator | Factors that promote engagement in smoking cessation (including quitting, abstinence). | Includes influence of the study and related materials |
| Red | Barrier | Factors that inhibit engagement in smoking cessation. | Factors external to the person |
| Red | Smoking motivations | Factors that propelled or continue to propel, participant to engage in smoking behaviors. | Internal/cognitive factors |
| Red | Smoking behaviors | Comments related to when, how much, and other factors of the participant's smoking behaviors. |  |
| Black | Other | Important or unusual comment that does not fit under any other code. |  |
| Yellow | Good quote |  |  |
| Yellow | HIV status | Comments related to how HIV status impacts smoking behaviors | Takes precedent over all other codes |

**Reminders to Coders**

- Aim for the smallest possible extract when coding while also retaining context
- Avoid double coding
- Not necessary to code age when participants began smoking
