# Supplementary material for: A qualitative investigation of tobacco use and cessation experiences among people living with HIV in Botswana
Source: Discov Public Health. 2026 Sep 18;23(1):1483. doi: 10.1186/s12982-026-02813-1 (PMC13588883; doi:10.1186/s12982-026-02813-1)
Supplement: Supplementary file 2 [file 12982_2026_2813_MOESM2_ESM.docx]

**Supplementary Materials 1: Tables of Responses to Consolidated Criteria for Reporting Qualitative Research**

| **Domain 1: Research team and reflexivity** | | |
| --- | --- | --- |
| **Personal Characteristics of Data Collectors** | | |
| 1. Interviewers | Who conducted the interviews? | 5 Research Assistants for the BSMART study. |
| 2. Credentials | What were their credentials? | 1. Degree in social work 2. Degree in economics and statistics 3. Diploma in health promotion and education 4. Certificate in HIV counseling and testing 5. Certificate in health care auxiliary |
| 3. Occupation | What was their occupation at the time of the study? | Research Assistants |
| 4. Gender | Were they a man or woman? | 3 were women and 2 were men |
| 5. Experience and training | What experience or training did they have? | All Research Assistants had previous experience with qualitative data collection prior to this role. For this role they also received additional training on research ethics, data storage and security, and conducting interviews. |
| **Relationship with Participants** | | |
| 6. Relationship established | Was a relationship established prior to study commencement. | Research Assistants had previously interacted with the participants in their role as Research Assistants for the BSMART study. They would have met the participant about 24 weeks prior to the interview. |
| 7. Participant knowledge of the interviewer | What did the participants know about them? | Most participants knew the interviewers as Research Assistants associated with the BSMART study and that their role was to support data collection. |
| 8. Interviewer characteristics | What characteristics were reported about the interviewer? | None |
| **Domain 2: Study design** | | |
| **Theoretical Framework** | | |
| 9. Methodological orientation and theory | What methodological orientation was stated to underpin the study? | Mixed deductive (based on interview guide) and inductive coding. |
| **Participant Selection** | | |
| 10. Sampling | How were participants selected? | Purposive sampling – we purposefully recruited to participants for these interviews during their Week 24 clinic visit. Participants were purposively sampled based on their smoking cessation behavior (i.e., quit, quit and resumed, or did not quit) stratified within their health facility. |
| 11. Method of approach | How were participants selected? | Purposive |
| 12. Sample size | How many participants were in the study? | 36 |
| 13. Non-participation | How many people refused to participate or dropped out? | 2 refused participating in the interviews |
| **Setting** | | |
| 14. Setting of data collection | Where was the data collected? | Interviews were conducted in a private space at the health facility pre-arranged to be available as part of the BSMART study. |
| 15. Presence of non-participants | Was anyone else present beside the participants and the researchers? | No. |
| 16. Description of sample | What are the important characteristics of the sample? | Smoking cessation behavior (i.e., quit, quit and resumed, or did not quit)  Health facility  Gender |
| **Data Collection** | | |
| 17. Interview guide | Were questions, prompts, guides provided by the authors? Was it pilot tested? | An interview guide was developed a priori and was not pilot tested. |
| 18. Repeat interviews | Were repeat interviews carried out? | No |
| 19. Audio/visual recording? | Did the research use audio or visual recordings to collect the data? | Audio recordings |
| 20. Field notes | Were field notes made during and/or after the interviews? | No |
| 21. Duration | What was the duration of the interviews? | 6-30 minutes |
| 22. Data saturation | Was data saturation discussed? | Yes, during data analysis saturation was discussed. On many aspects of the guide the team felt saturation was reached. |
| 23. Transcript returned | Were transcripts returned to participants for comments and/or correction? | No |
| **Domain 3: Analysis and findings** | | |
| **Data Analysis** | | |
| 24. Number of data coders | How many data coders coded the data? | 3 |
| 25. Description of the coding tree | Did authors provide a description of the coding tree? | Yes, codebook is provided in supplemental materials |
| 26. Derivation of themes | Were themes identified in advance or derived from the data? | Themes were derived from the data based on inductive and deductive coding. |
| 27. Software | What software, if applicable, was used to manage the data? | Transcripts were created in Microsoft Word.  Data was analyzed using ATLAS.ti |
| 28 Participant checking | Did participants provide feedback on the findings? | No |
| **Reporting** | | |
| 29. Quotations presented | Were participant quotations presented to illustrate the themes/findings? Was each quotation identified? | Yes, quotations were provided. Smoking cessation behavior was acknowledged. But individual participants were not identified. |
| 30. Data and finding consistent | Was there consistency between the data presented and the findings? | Yes |
| 31. Clarity of major themes | Were major themes clearly presented in the findings? | Yes |
| 32. Clarity of minor themes | Is there a description of diverse cases or discussion of minor themes? | Yes, inconsistencies or disagreements are described in the findings. |
