# Supplementary material for: A qualitative investigation of tobacco use and cessation experiences among people living with HIV in Botswana
Source: Discov Public Health. 2026 Sep 18;23(1):1483. doi: 10.1186/s12982-026-02813-1 (PMC13588883; doi:10.1186/s12982-026-02813-1)
Supplement: Supplementary file 3 [file 12982_2026_2813_MOESM3_ESM.docx]

**Supplementary Materials 2: Interview Guides**

Semi-Structured Interview Guides

Table of Contents

[Participants Who Did Not Quit Smoking	6](#_Toc222397009)

[Participants Who Quit then Resumed Smoking	11](#_Toc222397010)

[Participants Who are Currently not Smoking	16](#_Toc222397011)

# **Participants Who Did Not Quit Smoking**

***Note to Interviewer:***

**Text in red refers to important directions for you.**

**SECTION A: INTRODUCTION**

Thank you for taking the time to talk with me today. My name is ___________ and I am part of a team doing research on how we can help people quit smoking, especially when they encounter challenges when trying to do so.

Our conversation today will be quite informal, so please feel free to share anything you think would be important or helpful for us to know about your experiences with smoking and trying to quit.

I have a list of prepared questions that I will ask you. Sometimes I may ask you to expand or clarify your responses, I do this to make sure I fully understand. Other times I may ask that we move onto the next topic so that I can respect your time and ensure we can cover everything we need to get through.

You may or may not remember that I will be audio recording this interview. The reason for that is so that I can focus on what you are saying without trying to write it all down. No one will hear the recording except for people working on the project and we will remove any identifying information from the transcript of the interview. In reports from this project, we will never identify you as a participant or provide any information that allows other people to figure out you took part.

As a reminder, your participation in this interview is completely voluntary. If there are any questions you’d rather not answer, that is fine – just let me know. And, if at any point, you need a break, you can let me know that too. We are interested in your honest thoughts and experiences so there are no “right” or “wrong” answers to my questions.

Do you have any questions before we begin the interview?

[after responding to any questions or concerns] **START RECORDING**

[state participant ID, date, and approximate time]

Let’s get started!

**SECTION B: SMOKING EXPERIENCES**

First, I want to ask you a little about your history with smoking tobacco. Please, can you take a moment to think back to when you began smoking.

1. **How old were you when you started smoking regularly?**

*Prompt (if needed):*

If participant cannot give an exact age, a ballpark number or some other indicator (e.g., a year in school; before/after a significant life event) works as well

2. **What influenced you to begin smoking?**

*Prompt (if needed):*

- *What led you to start smoking?*
- *Did you feel pressured by anyone to smoke?*

Now thinking about over the last week

3. **Tell me about when and how much you smoke.**

*Prompt (if needed):*

- *What time of day?*
- *How many times on a typical day?*

4. **Does having HIV impact your smoking behaviors?**

*Prompt (if needed):*

- *Do you smoke more, less, or the same amount?*
- *Why do you think that is?*

5. **What makes you smoke?**

*Prompt (if needed):*

- *Pleasure, alleviate boredom*
- *Stress, manage depression or anxiety, manage anger*
- *Avoid withdrawal symptoms*
- *Manage pain or medications (e.g., make them work better, remove bad taste)*
- *Part of a social activity*
- *Weight management*
- *Need to (addicted to it); too hard to quit*
- *Avoid use of harder drugs or alcohol*
- *Helps with energy levels or concentration*
- *Not worried about health*

6. **Are these things that make you smoke now different from the things that made you smoke when you first started?**

*Prompt (if needed):*

- *Why do you think that is?*

Now, when you think about these reasons for why you smoke *[note a few or all of them]*

7. **Which of these reasons for smoking make it most difficult to quit?**

**SECTION C: SMOKING CESSATION**

Thank you for everything you have shared so far. I want to ask you a bit more about your thoughts and experiences with quitting smoking.

1. **Have you recently tried to quit smoking?** *IF YES,*
   a. **What reasons, recently, made you want to quit smoking?**
2. **Had you tried to quit smoking in the past? (Yes or No)**

*IF YES,* ***were you successful?***

*IF YES*

a. **What prompted you to quit smoking then?**
b. **What helped you the most with this?**
c. **Why do you think you weren’t able to successfully quit?**

*IF NO*

a. **Why do you think you have not tried to quit in the past?**
b. **What is different about your recent circumstances that you have tried to quit recently?**
3. **What characteristics or resources could help you quit next time?**

*Prompt (if needed):*

- *Aspects of your personality (e.g., determined, resilient)?*
- *Aspects of your situation (e.g., family or friends who motivate you)?*
- *How do these characteristics help you?*

4. **Is there anything else you think, had you had access to, would have helped you stop smoking?**

**SECTION E: CLOSING**

I really appreciate all of your answers and everything you have shared with me today.

1. **Is there anything else that you feel we should have talked about regarding your experiences with trying to quit smoking?**

Those are all the prepared questions I have. Thank you for taking the time to talk with me today. As a reminder, all of your responses will always be kept confidential and will never be linked with your name or other identifying information.

Do you have any questions for me at this time? If anything comes up in the future, your consent form has contact information listed on it. Please feel free to contact us.

[after responding to any questions or concerns] **STOP RECORDING**

# **Participants Who Quit then Resumed Smoking**

***Note to Interviewer:***

**Text in red refers to important directions for you.**

**SECTION A: INTRODUCTION**

Thank you for taking the time to talk with me today. My name is ___________ and I am part of a team doing research on how we can help people quit smoking, especially when they encounter challenges when trying to do so.

Our conversation today will be quite informal, so please feel free to share anything you think would be important or helpful for us to know about your experiences with smoking and trying to quit.

I have a list of prepared questions that I will ask you. Sometimes I may ask you to expand or clarify your responses, I do this to make sure I fully understand. Other times I may ask that we move onto the next topic so that I can respect your time and ensure we can cover everything we need to get through.

You may or may not remember that I will be audio recording this interview. The reason for that is so that I can focus on what you are saying without trying to write it all down. No one will hear the recording except for people working on the project and we will remove any identifying information from the transcript of the interview. In reports from this project, we will never identify you as a participant or provide any information that allows other people to figure out you took part.

As a reminder, your participation in this interview is completely voluntary. If there are any questions you’d rather not answer, that is fine – just let me know. And, if at any point, you need a break, you can let me know that too. We are interested in your honest thoughts and experiences so there are no “right” or “wrong” answers to my questions.

Do you have any questions before we begin the interview?

[after responding to any questions or concerns] **START RECORDING**

[state participant ID, date, and approximate time]

Let’s get started!

**SECTION B: SMOKING EXPERIENCES**

First, I want to ask you a little about your history with smoking tobacco. Please, can you take a moment to think back to when you began smoking.

8. **How old were you when you started smoking regularly?**

*Prompt (if needed):*

*If participant cannot give an exact age, a ballpark number or some other indicator (e.g., a year in school; before/after a significant life event) works as well.*

9. **What influenced you to begin smoking?**

*Prompt (if needed):*

- *What led you to start smoking?*
- *Did you feel pressured by anyone to smoke?*

Now thinking about over the last week

10. **Tell me about when and how much you used to smoke before you last stopped.**

*Prompt (if needed):*

- *What time of day?*
- *How many times on a typical day?*

11. **Does having HIV impact your smoking behaviors?**

*Prompt (if needed):*

- *Do you smoke more, less, or the same amount?*
- *Why do you think that is?*

12. **What makes you smoke lately?**

*Prompt (if needed):*

- *Pleasure, alleviate boredom*
- *Stress, manage depression or anxiety, manage anger*
- *Avoid withdrawal symptoms*
- *Manage pain or medications (e.g., make them work better, remove bad taste)*
- *Part of a social activity*
- *Weight management*
- *Need to (addicted to it); too hard to quit*
- *Avoid use of harder drugs or alcohol*
- *Helps with energy levels or concentration*
- *Not worried about health*

13. **Are these things that make you smoke now, different from the things that made you smoke when you first started?**

*Prompt (if needed):*

- *Why do you think that is?*

Now, when you think about these reasons for why you smoke *[note a few or all of them]*

14. **Which of these reasons for smoking made it most difficult for you to stay abstinent?**

**SECTION C: SMOKING CESSATION**

Thank you for everything you have shared so far. I want to ask you a bit more about your thoughts and experiences with quitting smoking.

5. **What reasons, recently, made you want to quit smoking?**
6. **Had you tried to quit smoking in the past?**

*IF YES*

a. What prompted you to quit smoking then?
b. What helped you the most with this?

**Now, thinking about your most recent attempt to quit smoking…**

c. What was different about your recent circumstances that made you try to quit recently?
d. Why do you think you weren’t able to stay abstinent this time?
e. How ready did you feel to quit?
f. When you decided to stop smoking again this most recent time, how confident were you that you could succeed?

7. **What characteristics or resources could help you stay abstinent next time?**

*Prompt (if needed):*

a. Aspects of your personality (e.g., determined, resilient)?
b. Aspects of your situation (e.g., family or friends who motivate you)?
c. How do these characteristics help you?

8. **Are there other things, beside your personal characteristics, that helped you resist smoking as long as you did?**

*Prompt (if needed):*

- Support from others (e.g., care team, family, friends); Medications
- Why do you think that was helpful?

9. **On the other hand, what challenges did you experience with staying abstinent?**
   a. What do you think would help you to overcome these challenges in the future?
10. **Is there anything else you think, had you had access to, would have helped you stay abstinent for longer?**

**SECTION E: CLOSING**

I really appreciate all of your answers and everything you have shared with me today.

2. **Is there anything else that you feel we should have talked about regarding your experiences with trying to quit smoking?**

Those are all the prepared questions I have. Thank you for taking the time to talk with me today. As a reminder, all of your responses will always be kept confidential and will never be linked with your name or other identifying information.

Do you have any questions for me at this time? If anything comes up in the future, your consent form has contact information listed on it. Please feel free to contact us.

[after responding to any questions or concerns] **STOP RECORDING**

# **Participants Who are Currently not Smoking**

***Note to Interviewer:***

**Text in red refers to important directions for you.**

**SECTION A: INTRODUCTION**

Thank you for taking the time to talk with me today. My name is ___________ and I am part of a team doing research on how we can help people quit smoking, especially when they encounter challenges when trying to do so.

Our conversation today will be quite informal, so please feel free to share anything you think would be important or helpful for us to know about your experiences with smoking and trying to quit. I have a list of prepared questions that I will ask you. Sometimes I may ask you to expand or clarify your responses, I do this to make sure I fully understand. Other times I may ask that we move onto the next topic so that I can respect your time and ensure we can cover everything we need to get through.

You may or may not remember that I will be audio recording this interview. The reason for that is so that I can focus on what you are saying without trying to write it all down. No one will hear the recording except for people working on the project and we will remove any identifying information from the transcript of the interview. In reports from this project, we will never identify you as a participant or provide any information that allows other people to figure out you took part.

As a reminder, your participation in this interview is completely voluntary. If there are any questions you’d rather not answer, that is fine – just let me know. And, if at any point, you need a break, you can let me know that too. We are interested in your honest thoughts and experiences so there are no “right” or “wrong” answers to my questions.

Do you have any questions before we begin the interview?

[after responding to any questions or concerns] **START RECORDING**

[state participant ID, date, and approximate time]

Let’s get started!

**SECTION B: SMOKING EXPERIENCES**

First, I want to ask you a little about your history with smoking tobacco. Please, can you take a moment to think back to when you began smoking.

15. **How old were you when you started smoking regularly?**

*Prompt (if needed):*

*If participant cannot give an exact age, a ballpark number or some other indicator (e.g., a year in school; before/after a significant life event) works as well*

16. **What influenced you to begin smoking?**

*Prompt (if needed):*

- *What led you to start smoking?*
- *Did you feel pressured by anyone to smoke?*

Now thinking about over the last week

17. **Tell me about when and how much you used to smoke before you last stopped?**

*Prompt (if needed):*

- *What time of day?*
- *How many times on a typical day?*

18. **When you smoked, did having HIV impact your smoking behaviors?**

*Prompt (if needed):*

- *Do you smoke more, less, or the same amount?*
- *Why do you think that is?*

19. **What made you smoke (before you stopped)?**

*Prompt (if needed):*

- *Pleasure, alleviate boredom*
- *Stress, manage depression or anxiety, manage anger*
- *Avoid withdrawal symptoms*
- *Manage pain or medications (e.g., make them work better, remove bad taste)*
- *Part of a social activity*
- *Weight management*
- *Need to (addicted to it); too hard to quit*
- *Avoid use of harder drugs or alcohol*
- *Helps with energy levels or concentration*
- *Not worried about health*

**SECTION C: SMOKING CESSATION**

Thank you for everything you have shared so far. I want to ask you a bit more about your thoughts and experiences with quitting smoking.

11. **What reasons, recently, made you want to quit smoking?**
12. **What helped you the most with this?**

Now, thinking about your most recent attempt to quit smoking…

13. **How ready did you feel to quit?**
14. **When you decided to stop smoking again this most recent time, how confident were you that you could succeed?**
    - *How capable did you feel?*
    - *How capable do you feel now?*
15. **What characteristics or resources do you have that you think helped you with quitting?**

*Prompt (if needed):*

- *Aspects of your personality (e.g., determined, resilient)?*
- *Aspects of your situation (e.g., family or friends who motivate you)?*
- *How do these characteristics help you?*

16. **Are there other things, beside your personal characteristics, that helped you quit and stay off smoking as long as you have?**

*Prompt (if needed):*

- *Support from others (e.g., care team, family, friends); Medications*
- *Why do you think that was helpful?*

17. **On the other hand, what challenges did you experience with quitting?**
    - *How did you overcome these challenges?*
18. **Is there anything else you think, had you had access to, would have helped you stop smoking sooner? Or would have made it easier for you to quit?**

**SECTION E: CLOSING**

I really appreciate all of your answers and everything you have shared with me today.

3. **Is there anything else that you feel we should have talked about regarding your experiences with quitting smoking?**

Those are all the prepared questions I have. Thank you for taking the time to talk with me today. As a reminder, all of your responses will always be kept confidential and will never be linked with your name or other identifying information.

Do you have any questions for me at this time? If anything comes up in the future, your consent form has contact information listed on it. Please feel free to contact us.

[after responding to any questions or concerns] **STOP RECORDING**
